# Correction: Multiple tumor suppressors regulate a HIF-dependent negative feedback loop via ISGF3 in human clear cell renal cancer

**DOI:** 10.7554/eLife.69256

**Published:** 2021-04-21

**Authors:** Lili Liao, Zongzhi Z Liu, Lauren Langbein, Weijia Cai, Eun-Ah Cho, Jie Na, Xiaohua Niu, Wei Jiang, Zhijiu Zhong, Wesley L Cai, Geetha Jagannathan, Essel Dulaimi, Joseph R Testa, Robert G Uzzo, Yuxin Wang, George R Stark, Jianxin Sun, Stephen C Peiper, Yaomin Xu, Qin Yan, Haifeng Yang

Liao L, Liu ZZ, Langbein L, Cai W, Cho E, Na J, Niu X, Jiang W, Zhong Z, Cai WL, Jagannathan G, Dulaimi E, Testa JR, Uzzo RG, Wang Y, Stark GR, Sun J, Peiper S, Xu Y, Yan Q, Yang H. 2018. Multiple tumor suppressors regulate a HIF-dependent negative feedback loop via ISGF3 in human clear cell renal cancer. *eLife* **7**:e37925. doi: 10.7554/eLife.37925.

Published 25, October 2018

We have been recently made aware through PubPeer of a mistake in Figure 5-figure supplement 1, where the anti-HA blots from Caki-1 and Ren-01 cells over-expressing HA-HIF2α-dPA appeared to be highly similar. After careful inspection of previous documents and consulting the original uncropped data, we had indeed mistakenly duplicated the band showing the anti-HA blot from the Ren-01. The error occurred when we copied and pasted the individual western blot results to create the figure supplement. The anti-HA blot from Ren-01 cells was likely distorted and duplicated during the figure creation process and mistakenly believed to be the anti-HA blot result of Caki-1 cells.

The correction does not affect the results or the conclusions of the original paper. However, it is an avoidable and regrettable mistake. We apologize for the mistake and the confusion this may have caused.

Corrected Figure 5-figure supplement 1:

**Figure 1:**
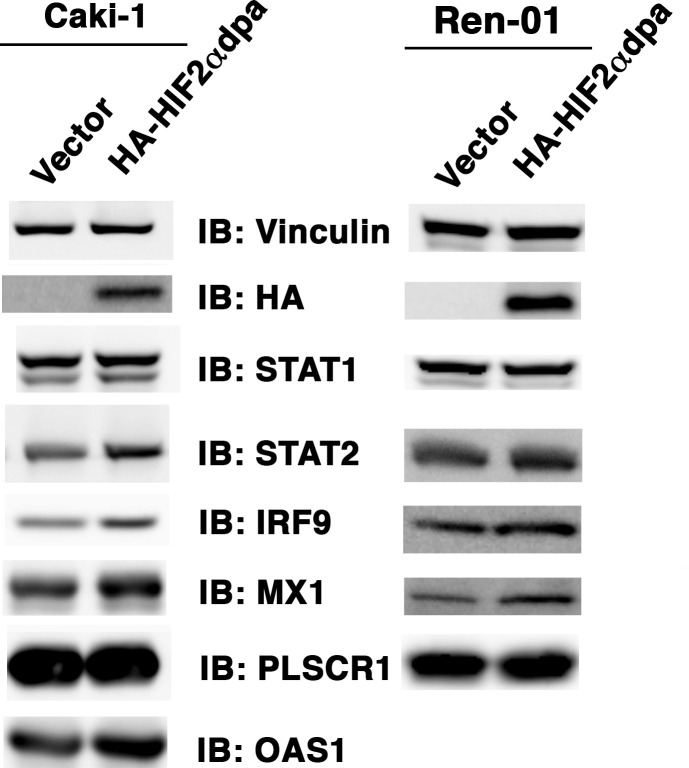


The original Figure 5—figure supplement 1 is shown here for reference:

**Figure 2:**
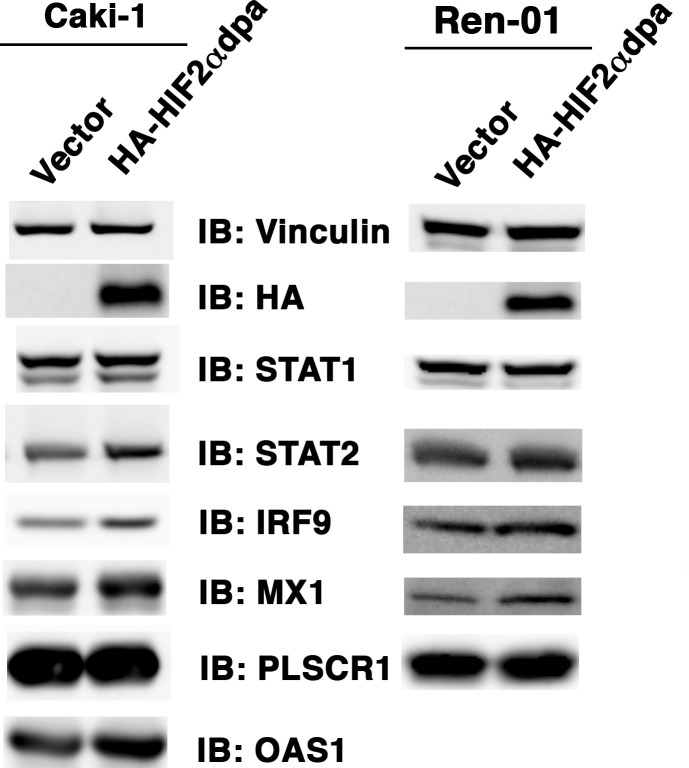


The article has been corrected accordingly.

