# Supplementary material for: Multiple tumor suppressors regulate a HIF-dependent negative feedback loop via ISGF3 in human clear cell renal cancer
Source: eLife. 2018 Oct 25;7:e37925. doi: 10.7554/eLife.37925 (PMC6234029; doi:10.7554/eLife.37925)
Supplement: Transparent reporting form [file elife-37925-transrepform.pdf]

eLIFE
1st Floor
24 Hills Road
Cambridge CB2 1JP, UK
P 01223 855340
W elifesciences.org
T @elife

eLife's transparent reporting form

We encourage authors to provide detailed information *within their submission* to facilitate the interpretation and replication of experiments. Authors can upload supporting documentation to indicate the use of appropriate reporting guidelines for health-related research (see EQUATOR Network), life science research (see the BioSharing Information Resource), or the ARRIVE guidelines for reporting work involving animal research. Where applicable, authors should refer to any relevant reporting standards documents in this form.

If you have any questions, please consult our Journal Policies and/or contact us: editorial@elifesciences.org.

Sample-size estimation

- You should state whether an appropriate sample size was computed when the study was being designed
- You should state the statistical method of sample size computation and any required assumptions
- If no explicit power analysis was used, you should describe how you decided what sample (replicate) size (number) to use

Please outline where this information can be found within the submission (e.g., sections or figure legends), or explain why this information doesn't apply to your submission:

The sample size is determined by the following analysis:

Sample size/power estimates (Table 1)
Table 1 shows the detectable difference between any two groups using either a 1 % or 5% level of significance and either 80% or 90% power, assuming the standard deviation within each group is 0.1 cc. These estimates are based on a two-sided t-test. As an example from this table, 80% power to detect a difference of 0.10 cc between any two groups using a 5% level of significance requires 17 animals per group in the study. **Ten animals per group provides 80% power to detect differences in tumor volume of 0.133 cc between any two groups (5% level of significance), and is considered acceptable for these studies.**

Note on significance level and power
When a statistical test is conducted it will be decided, based on the test, whether tumor volume differs between two groups. The significance level (or a) is the probability that two groups are different when they really are not. 100 minus the power is the probability that two groups are not different when they really are. Thus, if a statistical test has a 5% level of significance and 80% power, there is a 5% chance that tumor volume differs between two groups when it really does not, and a 20% chance that tumor volume does not differ between two groups when it actually does. For many studies, a 5% level of significance and 80% power are acceptable. Detectable differences for a 1 % level of significance and 90% power have also been included in the Table, so that detectable difference changes can be observed as these values change.

Table 1
Detectable Difference between Groups for
a Range of Powers and Sample Sizes*
*assumes S.D.=0.1cc a=significance level

	**80 % power**		**90 % power**	
**N/group**	**a=0.01**	**a=0.05**	**a=0.01**	**a=0.05**
5	0.268	0.202	0.301	0.234
6	0.234	0.179	0.262	0.208
7	0.210	0.163	0.234	0.189
8	0.192	0.151	0.216	0.174
9	0.178	0.141	0.201	0.163
10	0.167	0.133	0.188	0.153
11	0.158	0.126	0.178	0.145
12	0.150	0.120	0.169	0.139
13	0.143	0.115	0.161	0.133
14	0.137	0.110	0.155	0.127
15	0.132	0.106	0.149	0.123
16	0.127	0.102	0.144	0.119
17	0.123	0.099	0.139	0.115
18	0.119	0.096	0.135	0.111
19	0.116	0.093	0.131	0.108
20	0.113	0.091	0.127	0.105

* assumes S.D.=0.1 cc
a=significance level

The sample sizes are described in the figures.

Replicates

- You should report how often each experiment was performed
- You should include a definition of biological versus technical replication
- The data obtained should be provided and sufficient information should be provided to indicate the number of independent biological and/or technical replicates
- If you encountered any outliers, you should describe how these were handled
- Criteria for exclusion/inclusion of data should be clearly stated

- High-throughput sequence data should be uploaded before submission, with a private link for reviewers provided (these are available from both GEO and ArrayExpress)

Please outline where this information can be found within the submission (e.g., sections or figure legends), or explain why this information doesn't apply to your submission:

The mRNA samples used for microarray analyses were triplicates. The RT-PCR measurements were performed with duplicate and standard errors were calculated with two or three different experimental results. All the major western blot results were repeated one or two times, sometimes three times. Biological replicates indicate that the samples come from different experiments. Technical replicate repeat the measurement of the same biological samples.

The microarray results have been deposited into GEO. It is listed in materials and methods under gene expression analysis:
GEO 22 accession GSE108229: 23
Go to https://www.ncbi.nlm.nih.gov/geo/query/acc.cgi?acc=GSE108229 24
Enter token ermxqakmzhsrlqv into the box.

Statistical reporting

- Statistical analysis methods should be described and justified
- Raw data should be presented in figures whenever informative to do so (typically when N per group is less than 10)
- For each experiment, you should identify the statistical tests used, exact values of N, definitions of center, methods of multiple test correction, and dispersion and precision measures (e.g., mean, median, SD, SEM, confidence intervals; and, for the major substantive results, a measure of effect size (e.g., Pearson's r, Cohen's d)
- Report exact p-values wherever possible alongside the summary statistics and 95% confidence intervals. These should be reported for all key questions and not only when the p-value is less than 0.05.

Please outline where this information can be found within the submission (e.g., sections or figure legends), or explain why this information doesn't apply to your submission:

Statistical analysis methods and the calculation of p-values are described the materials and methods section.
Standard deviations and p values are provided in the figures and/or figure legends.

(For large datasets, or papers with a very large number of statistical tests, you may upload a single table file with tests, Ns, etc., with reference to sections in the manuscript.)

Group allocation

- Indicate how samples were allocated into experimental groups (in the case of clinical studies, please specify allocation to treatment method); if randomization was used, please also state if restricted randomization was applied
- Indicate if masking was used during group allocation, data collection and/or data analysis

Please outline where this information can be found within the submission (e.g., sections or figure legends), or explain why this information doesn't apply to your submission:

Not applicable

Additional data files ("source data")

- We encourage you to upload relevant additional data files, such as numerical data that are represented as a graph in a figure, or as a summary table
- Where provided, these should be in the most useful format, and they can be uploaded as "Source data" files linked to a main figure or table
- Include model definition files including the full list of parameters used
- Include code used for data analysis (e.g., R, MatLab)
- Avoid stating that data files are "available upon request"

Please indicate the figures or tables for which source data files have been provided:

For figures 1, 2, 3, 5 the source data has been provided in

GEO 22 accession GSE108229: 23
Go to https://www.ncbi.nlm.nih.gov/geo/query/acc.cgi?acc=GSE108229 24
Enter token ermxqakmzhsrlqv into the box.
